# Inhibition of Triple-Negative Breast Cancer Cell Aggressiveness by Cathepsin D Blockage: Role of Annexin A1

**DOI:** 10.3390/ijms20061337

**Published:** 2019-03-16

**Authors:** Mariana Alves Pereira Zóia, Fernanda Van Petten Azevedo, Lara Vecchi, Sara Teixeira Soares Mota, Vinícius de Rezende Rodovalho, Antonielle Oliveira Cordeiro, Lucas Ian Veloso Correia, Anielle Christine Almeida Silva, Veridiana de Melo Rodrigues Ávila, Thaise Gonçalves de Araújo, Luiz Ricardo Goulart

**Affiliations:** 1 Laboratory of Nanobiotechnology, Institute of Biotechnology, Federal University of Uberlandia, Uberlandia-MG 38400-902, Brazil; laravecchi7@yahoo.it (L.V.); thaisegaraujo@gmail.com (T.G.d.A.); 2 Laboratory of Biochemistry and Animal Toxins, Institute of Biotechnology, Federal University of Uberlandia, Uberlandia-MG 38400-902, Brazil; fvpetten@yahoo.com (F.V.P.A.); lucasian.veloso@gmail.com (L.I.V.C.); vmravila@hotmail.com (V.d.M.R.A.); 3 Laboratory of Genetics and Biotechnology, Institute of Biotechnology, Federal University of Uberlandia, Patos de Minas-MG 38700-128, Brazil; saratsm.s@hotmail.com (S.T.S.M.); antonielle._@hotmail.com (A.O.C.); 4 Laboratory of Biosensors, Institute of Biotechnology, Federal University of Uberlandia, Uberlandia-MG 38400-902, Brazil; vrrodovalho@gmail.com; 5 Laboratory of Isolation and Semiconductor Materials, Physics Institute, Federal University of Uberlandia, Uberlandia-MG 38408-902, Brazil; aniellechristineas@gmail.com; 6 Department of Medical Microbiology and Immunology, University of California Davis, Davis, CA 95616, USA

**Keywords:** Cathepsin D, Annexin A1, protease inhibition, triple-negative breast cancer

## Abstract

Triple-negative breast cancers (TNBCs) are more aggressive than other breast cancer (BC) subtypes and lack effective therapeutic options. Unraveling marker events of TNBCs may provide new directions for development of strategies for targeted TNBC therapy. Herein, we reported that Annexin A1 (AnxA1) and Cathepsin D (CatD) are highly expressed in MDA-MB-231 (TNBC lineage), compared to MCF-10A and MCF-7. Since the proposed concept was that CatD has protumorigenic activity associated with its ability to cleave AnxA1 (generating a 35.5 KDa fragment), we investigated this mechanism more deeply using the inhibitor of CatD, Pepstatin A (PepA). Fourier Transform Infrared (FTIR) spectroscopy demonstrated that PepA inhibits CatD activity by occupying its active site; the OH bond from PepA interacts with a CO bond from carboxylic acids of CatD catalytic aspartate dyad, favoring the deprotonation of Asp^33^ and consequently inhibiting CatD. Treatment of MDA-MB-231 cells with PepA induced apoptosis and autophagy processes while reducing the proliferation, invasion, and migration. Finally, in silico molecular docking demonstrated that the catalytic inhibition comprises Asp^231^ protonated and Asp^33^ deprotonated, proving all functional results obtained. Our findings elucidated critical CatD activity in TNBC cell trough AnxA1 cleavage, indicating the inhibition of CatD as a possible strategy for TNBC treatment.

## 1. Introduction

Breast cancer (BC) comprises a molecularly and clinically heterogeneous disease classified into multiple subtypes due to distinct biological features. Triple-negative breast cancer (TNBC) accounts for 15–20% of all diagnosed BCs and is a tumor type characterized by lack of expression of three markers: estrogen receptor (ER), progesterone receptor (PR), and human epidermal growth factor receptor 2 (HER2). This fact makes TNBC cells insensitive to hormone or HER2-targeted therapies; hence chemotherapy, surgery, and radiation therapy are established treatment options for TNBC patients. In this context, patients with TNBC have an unfavorable prognosis besides high risk of metastases, increased risk of tumor relapse, and worse survival rate, compared with other BC subtypes [1]. Thus, it is imperative to identify and characterize specific molecular events of the TNBC cell that could provide a rationale for innovative and efficient therapy for this subtype of BC.

Annexin A1 (AnxA1) is a 37 KDa protein that displays tumorigenic properties in TNBC and is potentially cleaved by Cathepsin D (CatD) solely at Trp^12^ at the N terminus, resulting in the generation of a cleaved isoform of AnxA1 (35.5 kDa) and of an N-terminal fragment [2]. Interestingly, the increased expression of cleaved AnxA1 reported in tumor cell lines and solid tumors suggests that this cleavage may be essential for maintenance of cancer aggressiveness [3,4].

Cathepsins are a superfamily of proteases including 16 members of aspartyl, serine, and cysteine proteases that are found highly expressed in various types of cancers and correlated with metastasis [5]. Especially in BC, CatD is an essential protease [6]. CatD is a lysosomal aspartic protease that is capable of cleaving several products such as myelin [7], insulin-like growth factor-binding proteins [8], hemoglobin [9], macrophage inflammatory proteins 1α (CCL3), 1β (CCL4), and SLC (CCL21) [10], collagen [11], glucagon [12], cholera enterotoxin [13], lipotropin [14], parathyroid hormone [15], and Annexin A1 [2] through the CatD active site that contains the catalytic aspartate dyad, specifically Asp^33^ and Asp^231^ [16]. Furthermore, CatD is explored as a TNBC marker. Despite the fact that the proteolytic events responsible for this BC subtype progression have not yet been elucidated, accumulating evidence indicates that high levels of CatD in TNBC primary tumors is indicative of local recurrence or distant metastasis. Moreover, CatD expression is suggested to be an independent prognostic factor for disease-free survival of TNBC patients [17,18]. We hypothesized that the protumorigenic activity of CatD involves its ability to generate the cleaved isoform of AnxA1.

Herein we have investigated an unexplored association between AnxA1 and CatD in TNBC and we succeeded in highlighting the molecular consequences of such interaction in the TNBC cell MDA-MB-231. We showed that MDA-MB-231 cells display upregulated levels of CatD and we demonstrated that blocking AnxA1 cleavage through CatD inhibition by Pepstatin A (PepA) is a critical mechanism for reducing the aggressiveness of these cells. Finally, this study sheds light, at the atomic level, on the mechanism of CatD active site inhibition to favor studies for drugs for several diseases whose development requires CatD.

## 2. Results

### 2.1. High Expression of AnxA1 Is Associated with CatD Expression in TNBC Cells

**Figure 1:**
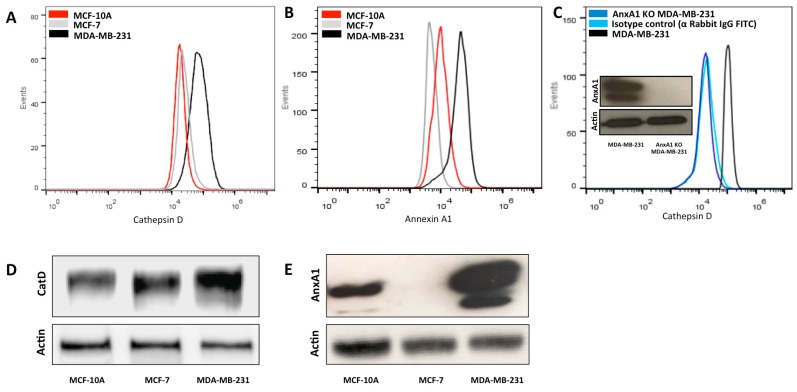
Enhanced expression of CatD in MDA-MB-231 cells is associated with AnxA1 expression. (**A**) Flow cytometry analysis of CatD overall protein expression in MCF-10A (red peak), MCF-7 (grey peak), and MDA-MB-231 (black peak) cells. (**B**) Flow cytometry analysis of AnxA1 overall protein expression in MCF-10A (red peak), MCF-7 (grey peak), and MDA-MB-231 (black peak) cells. (**C**) Flow cytometry analysis of CatD expression in MDA-MB-231 cells having native levels of AnxA1 (black peak), AnxA1 knockdown MDA-MB-231 (dark blue peak) cells in which AnxA1 expression was suppressed, and isotype control anti-Rabbit, IgG FITC (light blue peak). Western blotting analysis of AnxA1 expression of MDA-MB-231 cells having native levels and having complete knockdown of AnxA1 expression. (**D**) Western blotting analysis of CatD expression of MCF-10A, MCF-7, and MDA-MB-231 cells. Actin was used as loading control. (**E**) Western blot analysis of AnxA1 expression of MCF-10A, MCF-7, and MDA-MB-231 cells. Actin was used as loading control.

Previous studies have shown that AnxA1 upregulation predicts poor prognosis of BC [3], and is associated with cellular invasion in TNBC patients [3] and with resistance to chemotherapies in TNBC cells [19]. Additionally, overexpression of CatD in mammary tumors results in a poor survival rate and in aggressive metastasis [18]. In the present study, we have examined whether expression of AnxA1 is correlated to CatD expression in TNBC. We first used a flow cytometry assay and Western blotting assays to obtain the profile expression of both proteins in the three cell lines: MCF-10A (human mammary epithelial cell line, non-neoplastic), MCF-7 (non-TNBC, ER+/PR-HER2-), and MDA-MB-231 (TNBC). We demonstrated that the most aggressive of the three cell lines, namely MDA-MB-231, presents higher AnxA1 and CatD expression compared with MCF-10A and MCF-7 (Figure 1A–E). Consistent with these findings, the literature demonstrates that AnxA1 is highly expressed in MDA-MB-231, in samples from TNBC patients and in lymph node metastases in BC [3,20].

To clarify the effect of AnxA1 on CatD levels in TNBC cells, AnxA1 was stable knockdown in MDA-MB-231 cells using lentivirus-mediated shRNAs (this experiment was confirmed by Western blotting, Figure 1C). As shown in Figure 1C, CatD expression of the MDA-MB-231 AnxA1 knockdown cell line (AnxA1 KD MDA-MB-231) was lower than that of native MDA-MB-231 cells. These experiments indicated that AnxA1 and CatD act in concert in TNBC. An important question, which arose at this point, was how CatD modulates AnxA1. To address this, we used an inhibitor of CatD, PepA.

### 2.2. PepA Inhibts CatD through Carboxylic Acids from Catalytic Aspartate Dyad

In general, CatD inhibitors are being studied as a potential therapy for BC treatment [21]. In this context, PepA is a potent inhibitor of CatD activity [21]. To confirm the efficiency of CatD inhibition by PepA and to elucidate the atoms responsible for this, we have conducted FTIR assays. This technique enabled the characterization of the CatD-AnxA1 complex and the investigation of regions in the structures of CatD and PepA responsible for CatD inhibition, based on vibrations of atoms within molecules [22]. Since we have demonstrated that AnxA1 and CatD are correlated in TNBC, we performed this analysis only in the TNBC cell line.

**Figure 2:**
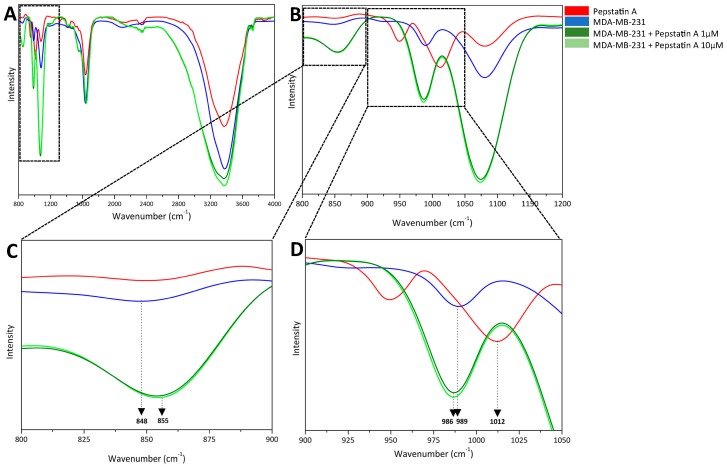
FTIR spectroscopy analysis of atoms involved in CatD inhibition. (**A**) Fourier transform infrared spectroscopy spectra of PepA (red), cells (blue), and cells treated with PepA (1 µM, light green; 10 µM, dark green) at a range of 800–4000 cm^−1^. For better visualization, spectra are zoomed in on the following ranges: (**B**) 800–1200 cm^−1^, (**C**) 800–900 cm^−1^ with dominant bands from OH bending vibration at 848 cm^−1^ and CO bending vibration at 855 cm^−1^, both from carboxylic acid from CatD aspartates; and (**D**) 900–1050 cm^−1^ with dominant bands at 986 cm^−1^ and 989 cm^−1^ from OH bending vibration and 1012 cm^−1^ from OH angular deformation from PepA molecule structure.

FTIR spectra of MDA-MB-231 cells, PepA, and MDA-MB-231 cells treated with PepA 1 μM and 10 μM in the frequency range of 800–4000 cm^−1^ are shown in Figure 2A. In the frequency range of 800–1200 cm^−1^ (Figure 2B), the spectrum was subjected to zooming for analysis of modifications; this figure was divided into two important regions: 800–900 cm^−1^ (Figure 2C) and 900–1050 cm^−1^ (Figure 2D) for better comprehension.

At first inspection, in the frequency range of 800–900 cm^−1^, the group containing only MDA-MB-231 cells is characterized by a band at 848 cm^−1^ attributed to OH bending vibration [23] originating from carboxylic oxygen atoms of protonated catalytic aspartate dyad from CatD. Then, in MDA-MB-231 cell + PepA 1 μM and 10 μM groups, inhibitor interacts with CatD from the TNBC cells and bands are found at 855 cm^−1^ attributed to CO bending vibration [24] from two catalytic aspartate residues from CatD. Taken together, these results indicate not only that carboxylic groups from catalytic aspartate dyad are involved in CatD inhibition by PepA, but also that these amino acids in the native protease are protonated. Moreover, as expected and supporting these observations, no PepA band was found in this experiment.

Furthermore, the frequency range of 900–1050 cm^−1^ confirms that PepA is able to inhibit CatD through its carboxylic group from the aspartate dyad. On one hand, bands at 986 cm^−1^ and 989 cm^−1^ from the groups of MDA-MB-231 cells treated with PepA 1 μM and 10 μM and the cells-only spectrum, respectively, are both from CO bending vibrations originating from the carboxylic group from CatD catalytic aspartate residues [25]. On the other hand, the band at 1012 cm^−1^ of the PepA spectrum was assigned to the angular deformation of the OH group [26] from PepA. Therefore, we can predict that PepA interacts with CatD through OH groups.

These data suggest three important events. First, in the native CatD, carboxylic acids from the CatD catalytic aspartate dyad are protonated. Second, the main atoms responsible for CatD inhibition are carboxylic acid, especially CO bond, from its active site and OH bond at PepA structure. Third, spectra of two groups (MDA-MB-231 + PepA 1 μM and MDA-MB-231 + PepA 10 μM) showed the same bands, meaning that PepA is able to effectively inhibit CatD at both concentrations tested.

### 2.3. CatD Cleaves AnxA1: A Crucial Event in TNBC Cells Proliferation and Invasion

A recent study suggests an association between AnxA1 cleavage and cancer aggressiveness and progression [27]. Although the literature highlights the role of CatD and cleaved AnxA1 in cancer, there are no studies correlating both in BC.

**Figure 3:**
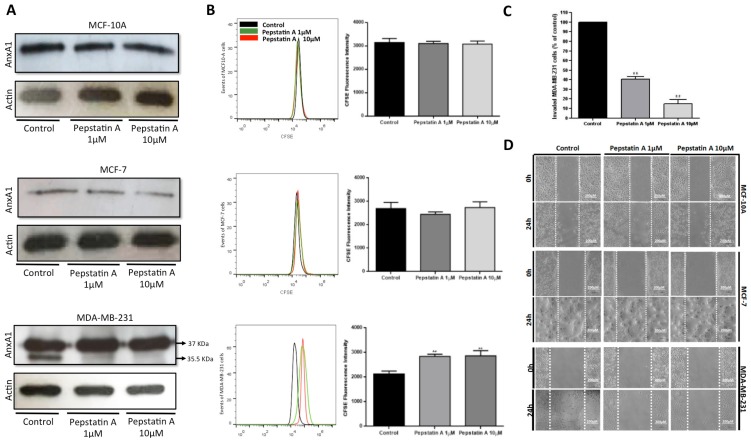
CatD inhibition by PepA impairs proliferation, migration, and invasion of MDA-MB-231. (**A**) Western blot analysis of AnxA1 expression of cells treated or not treated (control, vehicle only) for 24 h with 1 µM and 10 µM of PepA. Full-length AnxA1 (37 KDa) was detected in all cells and cleaved form (35.5 KDa) was detected only in MDA-MB-231 cells. Actin was used as loading control. (**B**) Proliferation analysis of MCF-10A, MCF-7, and MDA-MB-231 cells treated with PepA (1 µM, grey peak; and 10 µM, red peak) compared to control group (vehicle only, black peak) using CFSE staining. Respective bar graphs show the CFSE fluorescence intensity and statistical analysis of three independent experiments expressed as means ± SD (**, *p* < 0.01). (**C**) Percentage of invaded cells relative to control was measured by Matrigel invasion assay. Graph shows the percentage of invasion of three cell lines and statistical analysis of three independent experiments were expressed as means ± SD (**, *p* < 0.01). (**D**) Migration potential was assessed by wound-healing assay. Cells were plated, scratched with pipette tips, and photographed by phase-contrast microscopy. Representative images, showing cells migrated at 0 h and after 24 h. Scale bars = 200 µm.

Western blotting analysis of AnxA1 expression in the three lineages (Figure 3A) revealed higher expression of the full-length AnxA1 (37 KDa) in MDA-MB-231 cells compared with MCF-7 and MCF10-A cells. Cleaved AnxA1 (35.5 KDa fragment generated by CatD) was high and exclusively expressed in TNBC cells. We tested PepA at the respective concentrations of 1 and 10 μM in the three lineages. Interestingly, this drug caused an inhibition in the amount of cleaved AnxA1 in MDA-MB-231. Moreover, AnxA1 expression was not modified in MCF-10A and MCF-7 cells after PepA treatment since it was possible to visualize only the full-length form of Anxa1 in these cells. In fact, the AnxA1 35.5 KDa fragment, generated by CatD activity, was high and exclusively expressed in TNBC cells. Therefore, we investigated whether inhibition of CatD by PepA could suppress TN cell proliferation and invasion.

We first explored the effect of PepA on cell proliferation through CFSE staining. Results (Figure 3B) depict that treatment with PepA 1 μM and 10 μM concentrations for 24 h restricted only MDA-MB-231 proliferation, thus indicating that CatD and AnxA1 35.5 KDa are important for this process.

To analyze cell invasion after PepA treatment, we carried out a Matrigel invasion assay using the TNBC cell line treated with PepA (1 μM and 10 μM) for 24 h (Figure 3C). Compared to cells treated with vehicle only (control), relatively represented by 100% of invaded cells, PepA 1 μM decreased the percentage of MDA-MB-231 invasive cells to 40.75% and when treated with PepA 10 μM, only 15.00% of TNBC cell were able to invade the Matrigel. Thus, we found that the invasion ability of MDA-MB-231 cell line was decreased by PepA treatment. Finally, we verified whether CatD inhibition affects migration by means of the wound-healing assay. According to Figure 3D, PepA treatment did not diminish the migration ability of MCF-10A or MCF-7, but in TNBC cells, PepA decreased cell migration compared to the control.

Briefly, all these results indicate that CatD affects the aggressiveness of MDA-MB-231 cells through AnxA1 cleavage. It is known that AnxA1 autocrine signaling by its N-terminal peptide sustains proinvasive properties of melanoma cells [27]. We demonstrated that in BC, the blocking of AnxA1 cleavage is essential to reduce the proliferation, invasion, and migration properties of MDA-MB-231 cells as it prevents N-terminal peptides of this protein which elicit signaling pathways through FPR1 activation [3,27].

### 2.4. CatD Inhibition also Induces Apoptosis and Autophagy Processes in TNBC Cells

Since cleaved AnxA1 is highly expressed in MDA-MB-231 and required for the growth and survival of cancer cells, in this investigation we hypothesized that CatD may prevent apoptosis in TNBC. To explore whether CatD inhibition in MCF-10A, MCF-7, and MDA-MB-231 leads to apoptosis, cells were treated with PepA 1 μM and 10 μM for 24 h and further stained with Annexin V-PE and 7-AAD. Annexin V binds to cells in early apoptosis whereas the 7-AAD binds to such cells in late stages of cellular apoptosis.

**Figure 4:**
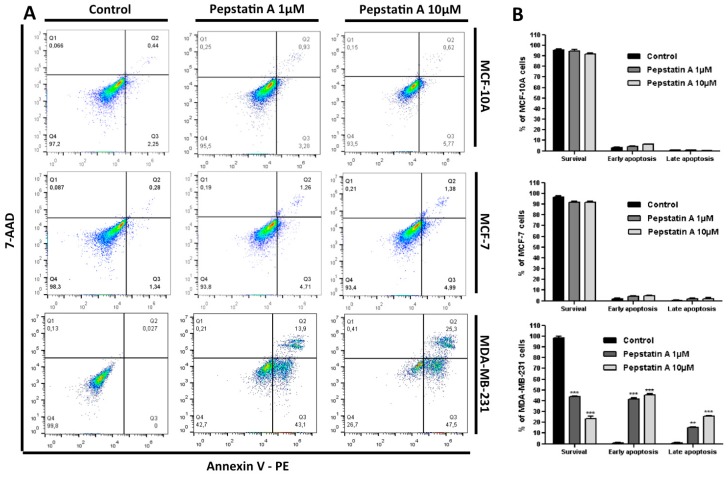
CatD inhibition induces apoptosis only in MDA-MB-231 cells. (**A**) The apoptotic fraction of MCF-10A, MCF-7, and MDA-MB-231 cells after 24 h treatment with PepA (1 µM and 10 µM) compared to control (vehicle only) is shown, with numerals in the lower right-hand panel (early apoptosis) and in the upper right-hand panel (late apoptosis). Annexin V and 7-AAD staining were used to assess the basal level of early (Annexin-V-labeled cells) and late apoptosis (Annexin-V- and 7-AAD-labeled cells). (**B**) PepA induced early and later apoptosis rate shown by histogram. Data are expressed as the mean ± SD of three independent experiments. Asterisks indicate significant difference from control group (** *p* < 0.01; *** *p* < 0.001). Comparisons between different groups were performed using one-way analysis of variance.

Flow cytometry investigation (Figure 4A) revealed that apoptosis was induced by PepA only in TNBC cells. The control MDA-MB-231 cells showed a viability percentage of 99.8% (Annexin V^−^/7-AAD^−^) (Figure 4B). However, after protease inhibition, the population of early apoptotic cells increased significantly (*p* < 0.001) from 0% to 43.1% (PepA 1 μM treatment) and to 47.5% (PepA 10 μM treatment). In relation to late apoptosis, we found that the percentage of double-positive Annexin V and 7-AAD cells increased significantly from 0.027%, in the control, to 13.9% (*p* < 0.05) and 25.3% (*p* < 0.001) among TNBC cells subjected to PepA 1 μM and 10 μM treatment, respectively. In contrast, CatD inhibition did not significantly contribute to apoptosis induction in MCF-10A and MCF-7 cells, in which no AnxA1 cleavage was found. These results indicate that CatD and the AnxA1 35.5 fragment can protect MDA-MB-231 cells from apoptosis and demonstrate that inhibition of AnxA1 cleavage, induced by CatD, promotes apoptotic cell death in 57% (PepA 1 μM) to 72.8% (PepA 10 μM) of TNBC cells.

Besides apoptosis, the programmed cell death known as autophagy has been reported to provide a backup mechanism to bypass resistance in apoptosis-refractory cells [28]. In addition, substantial evidence demonstrates that autophagy induction is a crucial event for drug-induced antitumor activity [29]. To investigate whether activation of apoptosis process by PepA is associated with an activation of autophagy in TNBC cells, we assessed autophagy by MDC staining. The fluorescent compound MDC is commonly employed to stain mature autophagic vacuoles, since it is a specific marker for autophagolysosomes [30].

**Figure 5:**
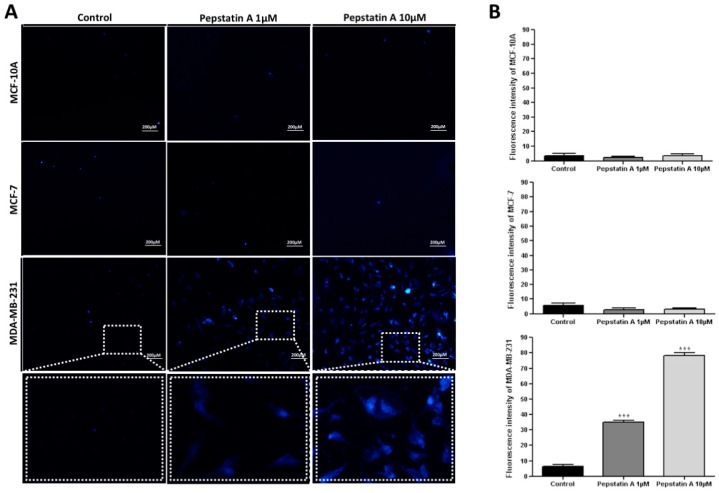
CatD inhibition promotes MDC-labeled autophagic vacuoles formation only in MDA-MB-231 cells. (**A**) Autophagic vacuoles (blue) in MCF-10A, MCF-7, and MDA-MB-231 cells stained with MDC after exposure to PepA (1 µM and 10 µM) for 24 h compared to control group (vehicle only). Dashed rectangle demonstrates the zoom view of each cell group. Scale bars = 200 µm. (**B**) Histogram representing fluorescence intensity of MDC, determined by ImageJ software, in control and treated three cell lines. Data are expressed as the mean ± SD of three independent determinations, *n* = 15. Asterisks (*) indicate significant difference from control group (* *p* < 0.05; ** *p* < 0.01; *** *p* < 0.001). Comparisons between different groups were performed using one-way analysis of variance.

In MCF-10A, MCF-7, and MDA-MB-231 control cells (treated with vehicle), MDC-labeled vacuoles were partially detected (Figure 5A). However, in TNBC cells treated for 24 h with PepA 1 μM and 10 μM, MDC-labeled vacuoles were detected with 35.00 and 78.40 fluorescence intensity, respectively (Figure 5B). Conversely, MCF-10A and MCF-7 cells did not present significant autophagic vacuole formation after PepA treatment, indicating that these cells are insensitive to CatD inhibition in relation to autophagy.

In agreement with the apoptosis results, these data demonstrated that AnxA1 35.5 KDa and CatD play a protective role against autophagy since PepA significantly induced this process in TNBC cells. Taken together, these results provide substantial evidence that CatD inhibition induced both apoptosis and autophagy processes in MDA-MB-231 cells. Recent studies [31,32,33] reveal that there is a form of death that is autophagy-dependent nonapoptotic, and based on this, our finding indicates that some percentage of apoptotic-resistant cells treated with PepA (43% in PepA 1 μM treatment and 27.2% in PepA 10 μM treatment) may have died in the autophagy process.

### 2.5. Validation of Structural Models of CatD/AnxA1 and CatD/PepA Interactions

The CatD/AnxA1 interaction allows AnxA1 cleavage, thus generating a 35.5 KDa fragment. When PepA blocks CatD (CatD/PepA complex), cleavage is inhibited, decreasing the aggressiveness potential of MDA-MB-231. Therefore, in order to assess the best conformation of the current studied complexes, in silico molecular docking studies were conducted.

**Figure 6:**
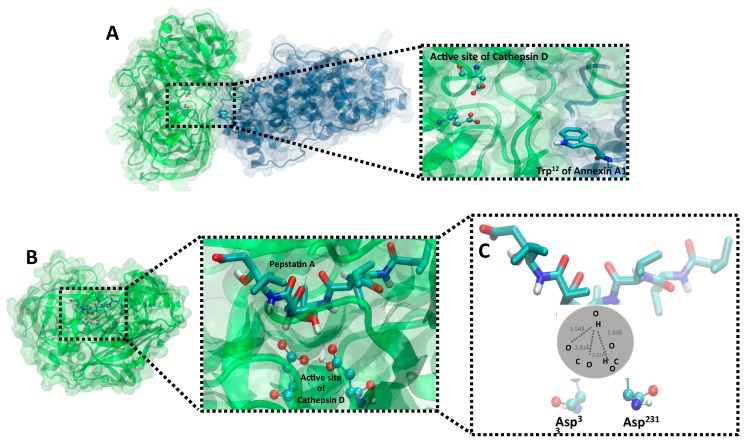
Molecular models of CatD/AnxA1 and CatD/PepA complexes. (**A**) Protein–protein docking of AnxA1 (blue) and CatD (green) interaction highlighting through dashed rectangle, on the zoom view, the active site (ball-and-stick model) of protease nearby AnxA1 Trp^12^ (licorice model). (**B**) A protein–ligand docking of CatD and PepA. A zoomed-in image, indicated by dashed lines, focusing on active site of CatD (ball-and-stick model) blocked by PepA (licorice model) is shown. All proteins are represented in cartoon model, whose spirals are alpha-helices and strips with arrows show beta-sheets, and transparent surface indicates the interface region. (**C**) A zoomed-in image at a different angle for better demonstration evidencing binding interactions in the PepA and CatD structure. OH of PepA has a hydrogen bond interaction with carboxylic acid of Asp^33^ and Asp^231^ (active site of CatD) with contact approximated distances of 3.54 Å, 2.83 Å, 2.832 Å, and 2.60 Å. Ball-and-stick and licorice models are colored by atom (nitrogen, dark blue; oxygen, red; hydrogen, white; carbon, light blue).

First, to analyze the CatD/AnxA1 complex, a protein–protein docking model was performed. The top cluster of resulting conformations presented 65 structures, the best of which presented a Haddock score of −161.6 ± 6.4, measured in arbitrary units, which considers intermolecular van der Waals, electrostatic, desolvation, and ambiguous interaction restraints energies [34]. It is known that CatD, when not inhibited by PepA, binds to AnxA1 at Trp^12^ [2] in a manner that approximates the catalytic site for cleavage and, accordingly, the position obtained from docking shows the proximity between Trp^12^ (AnxA1) and the catalytic aspartate dyad from CatD (Figure 6A). Nevertheless, by analyzing the conformation of CatD/AnxA1 docking, we speculate that there may be structural modifications not yet described which would expose more AnxA1 n-terminal domains, especially Trp^12^, to Asp^33^ and Asp^231^ to facilitate its cleavage.

In the second approach, to confirm the PepA activity and the main atoms involved in CatD inhibition predicted in the aforementioned experiments, protein–ligand docking (CatD/PepA complex) was conducted. The best cluster presented 36 PepA conformations while its most adequate structure showed a binding energy of −9.5353 kcal mol^−1^ with CatD, being structurally similar to the ligand conformation in the crystallography-determined complex [35]. As shown in Figure 6B, when CatD is inhibited by PepA, the inhibitor occupies the protease’s active site and, confirming FTIR findings, the suggested atoms (OH from PepA and carboxylic acid from CatD aspartate residues) are close. Moreover, in a zoomed-in view of the CatD/PepA complex (Figure 6C), docking demonstrated that Asp^231^ is protonated whereas Asp^33^ is not. This result was in accord with the inhibition mechanism of other aspartic proteases [36], such as the aspartic protease Human T-cell leukemia virus type 1 (HTLV-1) [37], in which the catalytic inhibition comprises one aspartate protonated and another deprotonated, both from the active site. In summary, at CatD’s active site, Asp^231^ is protonated, serving as an acid, while Asp^33^ is deprotonated, serving as a base, thus enabling CatD inhibition by OH from PepA.

## 3. Discussion

Although much effort has been put into the discovery of TNBC-targeted therapies, successes in discriminating the critical events that establish the characteristics of this most aggressive BC subtype have been limited. Thus, it is crucial to investigate critical mechanisms for TNBC to provide a guide for targeted therapies. Herein we show an association between AnxA1 and CatD, to report the successful exploitation of a TNBC cell marker event. Although CatD’s association with cancer is well known [5,6], this protein is not explored so far as a protease that could act in AnxA1 cleavage and we proved that this event is crucial for TNBC cell aggressiveness.

First, our findings demonstrated that CatD and AnxA1 are highly expressed in MDA-MB-231 cells, compared to MCF-10A and MCF-7 cells, and that the expression of these two proteins is associated. Previous studies indicated that CatD is an important biomarker among TNBC patients and that its high expression in advanced stages of BC results in tumor invasion and metastasis [18,21,38]. Additionally, it is known that AnxA1 is essential for aggressiveness of TNBC cells and the blocking of its signaling is considered a strategy for TNBC management [3]. Herein we provided the experimental data that correlate both proteins, demonstrating that CatD blockage inhibits AnxA1 cleavage. For that purpose, we used PepA.

We identified a previously unrecognized increased CatD expression in TNBC cells that results in 35.5 KDa AnxA1 fragment expression. These findings suggest that AnxA1 cleavage by CatD is a TNBC marker event and prompted us to examine the hypothesis that CatD inhibition by PepA is effective to inhibit AnxA1 cleavage and as a consequence, the aggressive behavior of TNBC cells. Controversial CatD inhibition effects are reported in BC. Some studies have proposed that PepA does not inhibit invasive behavior of MCF-7 [39]. In addition, substantial evidence suggests that this protease inhibitor had an effect on basement membrane degradation in MCF-7 and MDA-MB-231 [40,41]. We confirmed our hypothesis, demonstrating that CatD inhibition by PepA significantly enhanced not only TNBC cell apoptosis but also the autophagy process. Promotion of these two processes are important since recent studies suggest that some apoptosis-resistant cells may die upon activation of the autophagy process in a nonapoptotic cell-death mechanism [31,32,33].

Furthermore, our findings demonstrated that the blockage of AnxA1 cleavage resulted in diminution of TNBC cell proliferation, invasion, and migration. These in vitro findings pointed to a critical role of CatD in maintaining MDA-MB-231 aggressiveness. Besides these results, much evidence suggests that elevated CatD expression in tumors may protect cancer cells from chemotherapeutic agents [42] and, consequently, Pep A treatment could make cells more sensitive to chemotherapy.

As a proof of concept, we investigated the CatD catalytic activity mechanism by its inhibition through PepA to analyze the bonds and atoms involved in the CatD/PepA complex. In silico analysis demonstrated that PepA occupies the CatD active site, which contains Asp^33^ and Asp^231^ residues, thus hampering the proximity of CatD active site and Trp^12^ from AnxA1. Furthermore, FTIR analysis and molecular docking revealed that, on one hand, aspartate residues from the active site of native CatD are protonated. On the other hand, when the OH bond from PepA interacts with the CO bond from carboxylic acids of the catalytic aspartate dyad, Asp^231^ remains protonated while Asp^33^ becomes deprotonated, thus resulting in the inhibition of CatD. Taken together, these results elucidate the previously uncharacterized key atoms for CatD inhibition and provide a rationale that explains our functional results and could be exploited in further studies in order to develop CatD inhibitors with a highly effective inhibition mechanism. It is also worth noting that, although our focus was on TNBC treatment, the CatD inhibition information generated in this study also uncovered other potential applications in CatD-upregulated diseases, including the neurodegenerative disorders such as Alzheimer’s disease [43] and Parkinson’s disease [44], as well as atherosclerosis [45].

**Figure 7:**
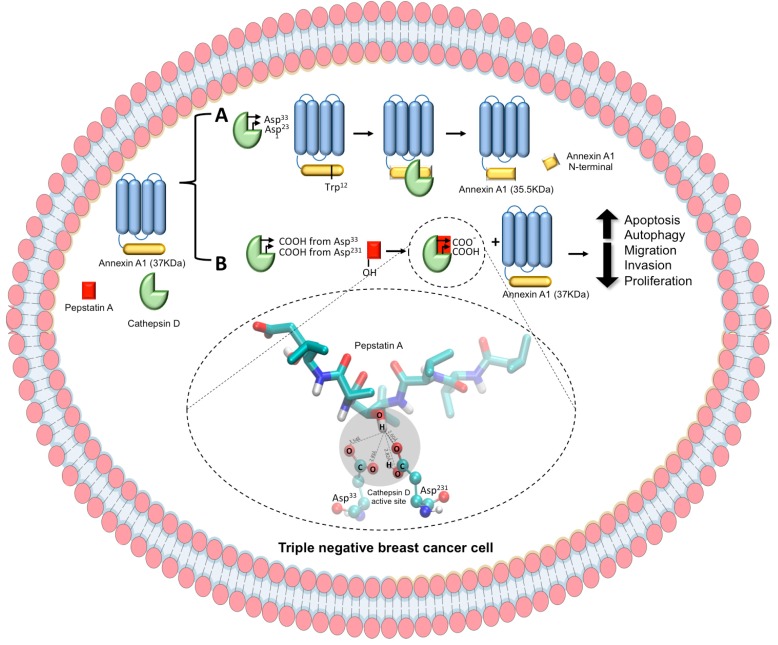
Schematic representation of CatD activity and inhibition by PepA and its in vitro consequences in TNBC cell. CatD is presented as a green generic representation, PepA, red square, and AnxA1 is presented as a core domain (blue) and an N-terminal domain (yellow). (**A**) On the one hand, CatD (with Asp^33^ and Asp^231^ from active site) cleaves AnxA1 at Trp^12^ to yield 35.5 KDa fragment. (**B**) On the other hand, OH from PepA interacts with carboxylic acids from Asp^33^ and Asp^231^ from CatD active sites, blocking CatD protease activity. Then, AnxA1 remains intact (37 KDa), leading to a decrease of MDA-MB-231 aggressiveness, which includes increase of apoptosis and autophagy process and decrease of migration, invasion, and proliferation rates.

In conclusion, in the present study on MDA-MB-231 cells under CatD blockage through PepA treatment, two molecular consequences were identified (see schematic diagram in Figure 7), namely (A) the CatD activity of cleaving AnxA1 in a 35.5 KDa fragment and (B) the effect of CatD inhibition on blocking AnxA1 cleavage, an effect associated with decreased TNBC cell aggressiveness. In addition, our findings elucidate CatD inhibition mechanisms that may enable advances in studies aimed at the development of an effective strategy for treatment of TNBC based on CatD inhibition once it plays a critical role on AnxA1 function.

## 4. Materials and Methods

### 4.1. Cell Culture

The nontumoral breast cell line, MCF-10A, and the breast tumor cells, MCF-7 and MDA-MB-231, were obtained from the American Type Culture Collection and cultured at 37 °C in a humidified atmosphere containing 5% CO_2_. MCF-10A cells were cultured employing DMEM F12 (Dulbecco’s Modified Eagle Medium: Nutrient Mixture F-12) (Life Technologies, Carlsbad, CA, USA) while MCF-7 and MDA-MB-231 cells were cultured using IMDM (Iscove’s Modified Dulbecco’s Media) (Life Technologies); both media were supplemented with 10% (*v*/*v*) fetal bovine serum and 1% (*v*/*v*) gentamicin. Cells were monitored by microscopy to maintain their original morphology. For starvation conditions, cells were incubated with serum-free medium for 24 h before the Western blot assay.

### 4.2. Generation of AnxA1 Knockdown MDA-MB-231 Cell Clones

We used commercially available lentiviral particles (Santa Cruz Biotechnology, Dallas, TX, USA) expressing either a control short hairpin (sh) RNA or an shRNA against AnxA1 to downregulate AnxA1 expression in TNBC cells. Lentiviral particles expressing shRNAs were transduced in MDA-MB-231 cells through a previous incubation with 5 µg/mL of Polybrene (Santa Cruz Biotechnology). Cells expressing the control shRNA or the shRNA against AnxA1 were selected by using 10 µg/mL of Puromycin (Santa Cruz Biotechnology).

### 4.3. Antibodies and Reagents

The antibodies used in this study were anti-AnxA1 (Invitrogen, Carlsbad, CA, USA), anti-CatD (Invitrogen, Carlsbad, CA, USA), and anti-Actin (Abcam). The following secondary antibodies were used: Horseradish peroxidase-conjugated goat anti-mouse IgG (Invitrogen) and goat anti-rabbit IgG (Sigma, St. Louis, MO, USA).

### 4.4. Protein Extraction and Western Blot

Total protein extracts of 24 h treated MCF-10A, MCF-7, and MDA-MB-231 cells in the absence (control cells, vehicle only) or presence of PepA (1 μM or 10 μM) were prepared using the NE-PER Nuclear and Cytoplasmic Extraction Reagent Kit (Thermo Scientific, Waltham, MA, USA). Protein concentration was determined by the BCA assay (Thermo Scientific). Equal amounts of cell proteins were separated by sodium dodecyl sulfate (SDS)-polyacrylamide gel electrophoresis (PAGE) after heating at 100 °C for 5 min. Proteins were transferred onto polyvinylidene difluoride (PVDF) membranes (Millipore, Billerica, MA, USA) and blocked for 1 h at room temperature in 5% skim milk. Next, the membranes were incubated for 1 h at room temperature with anti-Anxa1 antibody (rabbit polyclonal, 1:1000) and anti-Cathepsin D antibody (rabbit polyclonal, 1:1000), then incubated with horseradish peroxidase (HRP)-conjugated secondary antibody (anti-mouse, 1:3000, G-21040, Invitrogen; anti-rabbit IgG, 1:3000, G-21234, Invitrogen) for 1 h. Finally, blots were developed with the ECL Plus Kit (Amersham Biosciences, Corston, UK).

### 4.5. Apoptosis Assay

MCF10-A, MCF-7, and MDA-MB-231 cells (10^6^/well) were seeded in six-well culture plates and maintained at 37 °C, in 5% CO_2_, for 24 h. Subsequently, cells were incubated for 24 h with complete medium in the absence (control cells, vehicle only) or presence of PepA (1 μM or 10 μM). Then, cells were incubated with a specific binding buffer containing Annexin V-PE and 7-AAD (BD Pharmingen™, San Jose, CA, USA) for 30 min at 25 °C, protected from light. After incubation, the cells were analyzed by Flow Cytometry (BD Accuri C6, San Jose, CA, USA). The percentages of apoptotic cells was determined using the software FlowJo (Treestar, Inc., San Carlos, CA, USA).

### 4.6. Autophagy Assay

MCF10-A, MCF-7, and MDA-MB-231 cells were cultured on coverslips overnight and incubated with 1 μm or 10 μm of PepA for 24 h. Complete medium containing 50 μM of monodansylcadaverine (MDC) (Sigma) was incubated at 37 °C for 1 h. Subsequently, cells were washed with PBS and fixed for 15 min with ice-cold 4% paraformaldehyde at 4 °C, washed 3 times with glycerin, and analyzed by fluorescence microscopy (Zeiss LSM510, Dresden, Germany). Fluorescence intensity of MDC staining was measured by the software ImageJ. The final average fluorescence intensity per cell was calculated, and 15 cells were measured.

### 4.7. Proliferation Assay

To detect proliferation rates, MCF10-A, MCF-7, and MDA-MB-231 cells (10^6^/well), were labeled with 1 μM carboxyfluorescein diacetate (CFSE) (Invitrogen) for 10 min at 37 °C and subsequently cultivated in the presence or absence of PepA 1 μM or 10 μM. Treatments were applied to cells every other day for a total of five days. Finally, cell proliferation was measured using Flow Cytometry (Accuri C6, BD) followed by analysis by the software FlowJo.

### 4.8. Matrigel Invasion Assay

MCF10-A, MCF-7, and MDA-MB-231 were examined by the invasion assay by commercial BD BioCoat Matrigel 24-well Invasion Chambers, with 8.0 μm pore PET membrane (BD Pharmingen^TM^), according to the manufacturer’s instructions. Briefly, the chamber was rehydrated with serum-free medium for 2 h at 37 °C. Next, the chambers were placed in the lower compartment loaded with medium containing 5% fetal bovine serum (used as chemoattractant). Cells (2.5 × 10^5^/well) were seeded in the upper chamber in serum-free medium with PepA (10 uM) treatment or vehicle only (control cells). The cells were then allowed to invade through the Matrigel for 24 h at 37 °C and subsequently, invasive cells were fixed, stained, and counted manually under the microscope (EVOS FL; AMG).

### 4.9. Wound-Healing Migration Assay

Cell migration was examined by the wound-healing assay. The MCF10-A, MCF-7, and MDA-MB-231 cells (2 × 10^5^ cells/well) were seeded onto 24-well culture plates and maintained at 37 °C in 5% CO_2_ overnight. After complete confluence was achieved, the culture cell monolayers were scratched using a plastic pipette tip. Then, the cell monolayers were incubated for 24 h with medium in the absence (control cells, vehicle only) or presence of PepA (1 μM or 10 μM). The cellular confluence was analyzed in an inverted optical microscope (Nikon Eclipse TS100, Melville, NY, USA).

### 4.10. FTIR Analysis

The infrared (IR) spectra were recorded at room temperature using a Fourier Transform IR (FTIR) spectrophotometer via a total attenuated reflectance (ATR) at resolution 2 cm^−1^. The baseline correction was applied. Measurements were performed in the scanning range of 4000–400 cm^−1^. The FTIR spectra of the MDA-MB-231 cells treated with PepA (1 μM or 10 μM) were compared with MDA-MB-231 cells and PepA alone. Samples were applied in triplicate, randomly, permitting possible variations within or between plates to be taken into account during analysis. Loaded sample plates were oven-dried to remove extraneous moisture prior to FTIR analysis.

### 4.11. Molecular Modeling Docking Analysis

Three-dimensional structures of human CatD and PepA were obtained from the Protein Data Bank (PDB ID: 1LYA, ILYB) [35], whereas the protonation states of residues were predicted with PDB2PQR Server [46]. The AnxA1 three-dimensional structure was predicted using I-Tasser suite [47,48,49], its amino acid sequence was retrieved from UniProtKB (P04083), while a structure from PDB (PDB ID: 1HM6) [50] was used as a template.

The CatD was separately docked with AnxA1 and PepA. Protein–protein docking (CatD/AnxA1 complex) was carried out using the Haddock web server [51], whereas protein–ligand docking (CatD/PepA complex) was conducted with Autodock4 and AutoDockTools4 [51]. Docking analysis was performed according to the literature protocols [52] and experimental data from protein interactions. The docking calculations were performed in accordance with molecular docking protocols [52] and interactions between proteins. Interactions identification was performed with the web interface Arpeggio [53] and final images were prepared with Visual Molecular Dynamics (VMD) [54].

### 4.12. Statistical Analysis

All results are representative of at least three different experiments. Data were reported as the mean value ± standard deviation (SD). The statistical significance of the difference between groups was determined by the Student’s *t* test and was considered significant when *p* < 0.05. The statistical analyses were performed by using GraphPad Prism 7 (GraphPad Software Inc., La Jolla, CA, USA).

## 5. Conclusions

In this work, we have demonstrated that CatD is differentially expressed in the human TNBC cell line, in which Annexin A1 (AnxA1) is cleaved, an event that is exploited in this proposed concept as a critical mechanism for TNBC aggressiveness. Thereby, we elucidated at the atomic level the CatD inhibition mechanism and demonstrated that CatD inhibition by PepA plays a role in decreasing TNBC cell aggressiveness. We envision that the CatD inhibition mechanism will not only be applied for therapeutic strategies against TNBC cells, but will also be useful for designing improved inhibitors and for treatment of other CatD-associated diseases.

## Abbreviations

AnxA1	Annexin A1
BC	Breast cancer
CatD	Cathepsin D
CFSE	Carboxylfluorescein succinimidyl ester
ER	Estrogen receptor
FTIR	Fourier transform infrared
HER2	Human epidermal growth factor receptor 2
MDC	Monodansylcadaverine
PepA	Pepstatin A
PR	Progesterone receptor
TNBC	Triple-negative breast cancer
